# Novel Strain *Leuconostoc lactis* DMLL10 from Traditional Korean Fermented *Kimchi* as a Starter Candidate for Fermented Foods

**DOI:** 10.4014/jmb.2306.06056

**Published:** 2023-09-07

**Authors:** Yura Moon, Sojeong Heo, Hee-Jung Park, Hae Woong Park, Do-Won Jeong

**Affiliations:** 1 Department of Food and Nutrition, Dongduk Women’s University, Seoul 02748, Republic of Korea; 2 Department of Food and Nutrition, Sangmyung University, Seoul 03016, Republic of Korea; 3 Technology Innovation Research Division, World Institute of Kimchi, Gwangju 61755, Republic of Korea

**Keywords:** *Leuconostoc lactis* strain DMLL10, *Kimchi*, starter, genome, safety, enzyme

## Abstract

*Leuconostoc lactis* strain DMLL10 was isolated from *kimchi*, a fermented vegetable, as a starter candidate through safety and technological assessments. Strain DMLL10 was susceptible to ampicillin, chloramphenicol, clindamycin, erythromycin, gentamicin, kanamycin, streptomycin, and tetracycline. It did not show any hemolytic activity. Regarding its phenotypic results related to its safety properties, genomic analysis revealed that strain DMLL10 did not encode for any toxin genes such as hemolysin found in the same genus. It did not acquire antibiotic resistance genes either. Strain DMLL10 showed protease activity on agar containing NaCl up to 3%. The genome of DMLL10 encoded for protease genes and possessed genes associated with hetero- and homo-lactic fermentative pathways for lactate production. Finally, strain DMLL10 showed antibacterial activity against seven common foodborne pathogens, although bacteriocin genes were not identified from its genome. These results indicates that strain DMLL10 is a novel starter candidate with safety, enzyme activity, and bacteriocin activity. The complete genomic sequence of DMLL10 will contribute to our understanding of the genetic basis of probiotic properties and allow for assessment of the effectiveness of this strain as a starter or probiotic for use in the food industry.

## Introduction

*Leuconostoc* is a genus of Gram-positive bacteria that are ubiquitously distributed in various environments including plant, human clinical sources, and a variety of foodstuffs such as fermented meats, fermented vegetables, and fermented dairy products (*e.g.*, cheese) [1-6]. *Leuconostoc* spp. are generally known as non-pathogenic bacteria recognized as safe. They are rarely isolated from human clinical source [7]. *Leuconostoc* spp. are also well-known hetero-lactic fermentative bacteria that contribute to nutritional and sensory properties of fermented foods [3, 7].

*Leuconostoc lactis* is a Gram-positive, catalase negative, facultative anaerobic, and non-spore-forming lactic acid bacterium (LAB). It is mainly isolated from various environments such as cheese, whey, and *kimchi* [3, 8, 9]. *Leuconostoc lactis* is a hetero-lactic fermentative bacterium that produces equimolar of lactate, ethanol, and carbon dioxide from one mole of glucose in the absence of oxygen, while it produces acetate instead of lactate in the presence of oxygen [10]. Some strains of *Leu. lactis* produce butter-flavored products such as diacetyl and acetoin at low pH. They can convert carbohydrates such as sucrose into dextran exopolysaccharides [2, 11, 12]. Metabolic products produced by *Leu. lactis* play an important role in storage and nutrition of fermented foods, making them suitable as fermentation bacteria.

*Leuconostoc lactis* has been reported to be nonpathogenic. It is on the Qualified Presumption of Safety (QPS) list of the European Union Food Safety Authority (EFSA) [13]. *Leu. lactis* is also on the International Dairy Federation (IDF) list as a fermented species of dairy products and alcoholic beverages [14-17]. The above results are sufficient to assume *Leu. lactis* is a safe species. However, *Leu. lactis* is not on the Food Materials list of the Ministry of Food and Drug Safety, Korea as of December 2022 unfortunately. Even if it is not registered as a food raw material in Korea, the same kind of substance registered with IDF or Generally Recognized as Safe (GRAS) can be used for that purpose. In other words, *Leu. lactis* is available as a dairy starter in Korea according to results of IDF. However, it is currently not available for vegetable fermentation such as *kimchi*. If you want to use it as a vegetable fermentation in Korea, *Leu. lactis* must be registered as a temporary food ingredient accompanied by a toxicity assessment for registration. For this reason, in Korea, *Leu. lactis* is limited as a starter species of fermented food.

*Kimchi* is the generic term given to a group of fermented vegetable foods in Korea [18]. Fermentation of traditional fermented *kimchi* depends on indigenous microflora, while commercial *kimchi* has been fermented by starter culture using *Leuconostoc mesenteroides* in most cases. Reasons for using a starter culture are: 1) the quality of mass-produced *kimchi* is maintained, 2) the fermentation period is shortened, and 3) the edible period is extended. It is known that the quality of fermentation caused by unwanted bacteria causes economic loss [19-21]. Until now, research has been focused on *Leu. mesenteroides* as a starter for *kimchi* fermentation. As a result, commercial *kimchi* using *Leu. mesenteroides* has been produced and sold. Although *Leu. lactis* is a member of the same genus as *Leu. mesenteroides* and frequently detected in *kimchi*, research on *Leu. lactis* as a starter candidate for *kimchi* is insufficient. It is recommended to have a large pool of starter candidates because the starter should contribute to producing suitable end products according to its purpose. Therefore, this study reports the isolation of a novel starter candidate, *Leu. lactis* DMLL10, from *kimchi*. Additionally, the safety and technological activity of *Leu. lactis* DMLL10 were confirmed not only by phenotyping, but also by genome analysis.

## Materials and Methods

### Bacterial Strains and Culture Conditions

*Leuconostoc lactis* DMLL10, originally isolated from *Jeonbok-baechu-kimchi*, was selected as a novel starter candidate and subjected to in vitro experiments and genomic analysis. *Leuconostoc lactis* KCTC 3528^T^ was used to compare phenotypical properties as the same species. *Leu. lactis* strains were cultured in Lactobacilli MRS broth (Becton, Dickinson and Co., USA) at 30°C for 18 h to maintain bacterial traits.

### Genome Sequencing

Genomic DNA was isolated and purified using a MagAttract HMW DNA Kit (Qiagen, Germany). The concentration and purity of extracted DNA were determined using a Qubit 2.0 fluorometer (Invitrogen, USA). Whole-genome sequencing of strain DMLL10 was performed using Single-Molecule Real-Time (SMRT) sequencing system (10 kbp) on a PacBio Sequel platform (Pacific Bioscience, USA) by CJ Bioscience, Inc. (Korea). A total of 136,666 reads (5355.39 × coverage) were generated. These reads were assembled into one contig using CLC Genomics Workbench ver. 7.5.1(CLC Bio, Denmark) with the HGAP4 algorithm in SMRT Link (version 10.1.0; Pacific Bioscience). Genome annotation was performed using the NCBI Prokaryotic Genome Annotation Pipeline (version 4.6) [22]. Open Reading Frames (ORFs) were predicted using Glimmer 3 [23], followed by annotation through a search against Clusters of Orthologous Groups (COG) database [23].

### Comparative Genomics

For genome comparison, genomes of type strain (KCTC 3528T) KCTC 3528^T^ from milk (GenBank Accession No. AEOR01000000) and four strains from fermented *kimchi*, CBA3622 (CP042420-CP042423), CBA3625 (CP042387-CP042388), CBA3626 (CP042390-CP042391), and strain WiKim40 (CP016598-CP016601), were used (Table 1). Genome sequence data were retrieved from the National Center for Biotechnology Information (NCBI) database (http://ncbi.nlm.nih.gov/genomes). Average Nucleotide Identity (ANI) was used to check similarity of the core genome [24]. Core-genome and pan-genome analyses were performed using the Efficient Database framework for comparative Genome Analyses using BLASTP score Ratios (EDGAR) [25]. Rapid Annotation using Subsystem Technology (RAST) [26] and Interactive Pathways Explorer v3 (https://pathways.embl.de/) were used to determine gene contents based on functional subsystem classifications and estimate metabolic pathways. Comparative analyses at protein level were performed by an all-against-all comparison of annotated genomes.

### Antibiotic Minimum Inhibitory Concentrations Analysis

Antibiotic Minimum Inhibitory Concentrations (MICs) were determined by the broth microdilution method [27]. Antibiotics was prepared with serial two-fold dilutions in deionized water. The final concentration of each antibiotic in a 96-microwell plate ranged from 0.5 mg/l to 32 mg/l. Bacterial strains were cultured twice in MRS broth and matched to a 0.5 McFarland turbidity standard (bioMérieux, France). Each suspension was further diluted 1:100 in cation-adjusted Mueller-Hinton broth (Becton, Dickinson and Co.) supplemented with 5% (v/v) sheep blood (MB Cell, Korea) to achieve an appropriate inoculum concentration. The final inoculum density was 5×10^5^ colony-forming units/ml. The inoculum (200 μl) was then added to each well of the 96-microwell plate. MICs of eight antibiotics were recorded as the lowest concentrations where no growth was observed in wells after incubation at 30°C for 18 h. MIC results were confirmed by at least three independently performed tests. All experiments were conducted at least three times on separate days. Strains with MICs higher than the breakpoint were considered resistant [28].

### Hemolytic Activity Tests

Tryptic Soy Agar (TSA; Becton, Dickinson and Co.) supplemented with 5% (v/v) rabbit blood (MB Cell) or 5%(v/v) sheep blood was used for α- or β-hemolytic activity test, respectively. The α-hemolytic activity was determined by incubation at 30°C for 24 h and the β-hemolytic activity was determined by cold shock at 4°C for 24 h after incubation at 30°C for 24 h [29]. Hemolytic activities were determined by formation of clear lytic zones around colonies on each blood-containing TSA plate. *Staphylococcus aureus* USA300-p23 and RN4220 were used as positive and negative controls, respectively, for hemolytic analyses [30]. All experiments were conducted at least three times on separate days.

### Acid Production and Enzymatic Activity

Acid production was determined on TSA containing 0.5% (w/v) glucose and 0.7% (w/v) CaCO_3_. Protease activity was determined on TSA containing 0.5% (w/v) glucose and 2% (w/v) skim milk. Lipase activity was tested on tributyrin agar (Sigma-Aldrich, USA) containing 1% (v/v) tributyrin and 0.5% (w/v) glucose. The tributyrin-supplemented medium was emulsified by sonication before autoclaving. To check enzymatic activity, filter paper discs were placed on each substrate-supplemented agar medium surface and 10 μl of *Leu. lactis* cultured on MRS broth was dropped onto these filter paper discs. Substrate-supplemented agar plates were then incubated at 30°C for 18 h. The relative size of the zone of clearing around the filter paper disc was used as an indicator of enzymatic activity. The effect of NaCl on protease activity was determined by adding NaCl to each medium up to a final concentration of 6% (w/v). All experiments were conducted at least two times on separate days.

### Determination of Bacteriocin Activity

Antibacterial activities of strain DMLL10 against nine foodborne pathogenic bacteria (*Bacillus cereus* KCCM 11341, *Enterococcus faecalis* KCTC 2011, *Listeria monocytogenes* ATCC 19111, *Staphylococcus aureus* ATCC 12692, *Alcaligenes xylosoxidans* KCCM 40240, *Flavobacterium* sp. KCCM 11374, *Escherichia coli* O157:H7 EDL 933, *Vibrio parahaemolyticus* KCTC 2729, and *Salmonella enterica* KCCM 11862) were determined using the agar well diffusion method. Pathogens as indicator strains from overnight culture in TSB (Becton, Dickinson and Co.) were inoculated at 1% (v/v) into fresh TSB and incubated to an OD_600_ of 1.0 and 200 μl of each culture was then poured onto TSA. A hole with a diameter of 6 mm was punched aseptically with a sterile cork borer and a 50 μl of concentrated supernatant of *Leu. lactis* was introduced into the well. These agar plates were then incubated at 30°C for 18 h. The concentrated supernatant of *Leu. lactis* was obtained from culture after incubating the culture at 30°C in MRS broth for 24 h: the supernatant was then obtained through centrifugation followed by concentration four times using a HyperVAC (Centrifugal Vacuum Concentrator VC2124, Hanil Scientific Inc., Korea). The relative size of the zone of clearing around the punched hole was used as an indicator of antibacterial activity. All experiments were conducted at least two times on separate days.

### Statistical Analysis

Duncan’s multiple range test following a one-way analysis of variance (ANOVA) was used to evaluate significant differences between average values of enzymatic and antimicrobial activities. Values with *p* < 0.05 were considered statistically significant. All statistical analysis was per-formed using the SPSS software package (version 27.0; SPSS, IBM, USA).

### Nucleotide Sequence Accession Number

The complete genome sequence of *Leu. lactis* DMLL10 was deposited in DDBJ/ENA/GenBank (Accession No. CP116456) and the Korean Culture Center of Microorganisms (Accession No. KFCC11941P).

## Results and Discussion

### Genetic Information of *Leuconostoc lactis* DMLL10

**Fig. 1:**
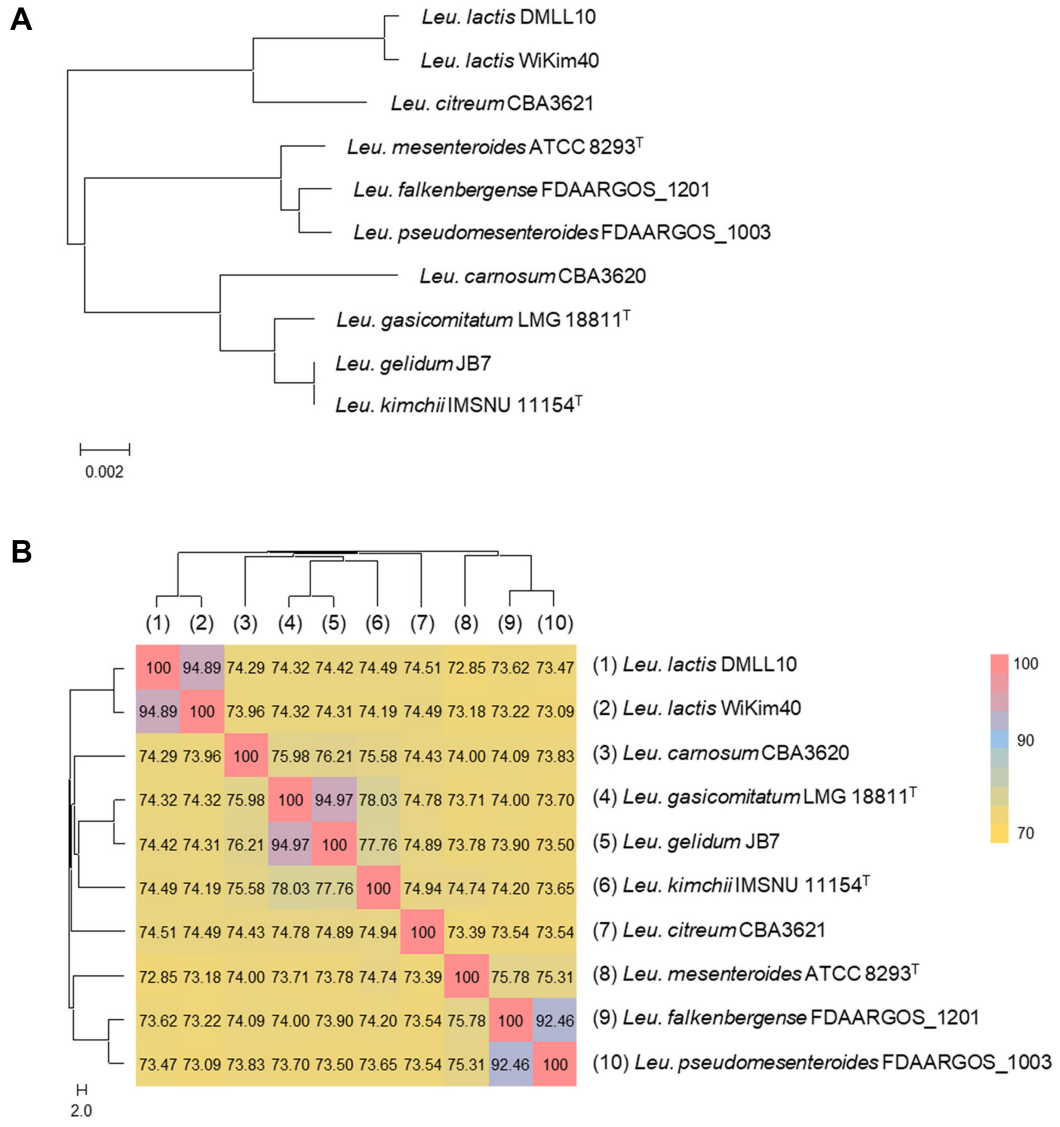
Phylogenetic analysis of *Leuconostoc lactis* DMLL10 based on (A) 16S rRNA gene sequences and (B) average nucleotide identity. Data were compared using simple matching coefficients and clustered by the maximum likelihood method. Branches with bootstrap values of 50% are collapsed. The scale of the diagram is pairwise distance expressed as percentage of dissimilarity.

Strain DMLL10 was isolated from *Jeonbok-baechu-kimchi*. The 16S rRNA sequence of strain DMLL10 showed 99.9% identity with the other *Leu. lactis* strain (WiKim40) and distinguished from other species (Fig. 1A). The 16S rRNA similarity among other species was determined to be higher than 97.5%, exceeding the 16S rRNA similarity threshold of 97% for species classification. In ANI analysis using genomic sequences, the DMLL10 genomic sequence shared 94.89% similarities with *Leu. lactis* WiKim40 (Fig. 1B). ANI analysis also showed clear discrimination from other species. These results could also be found in the phylogenetic tree.

**Fig. 2:**
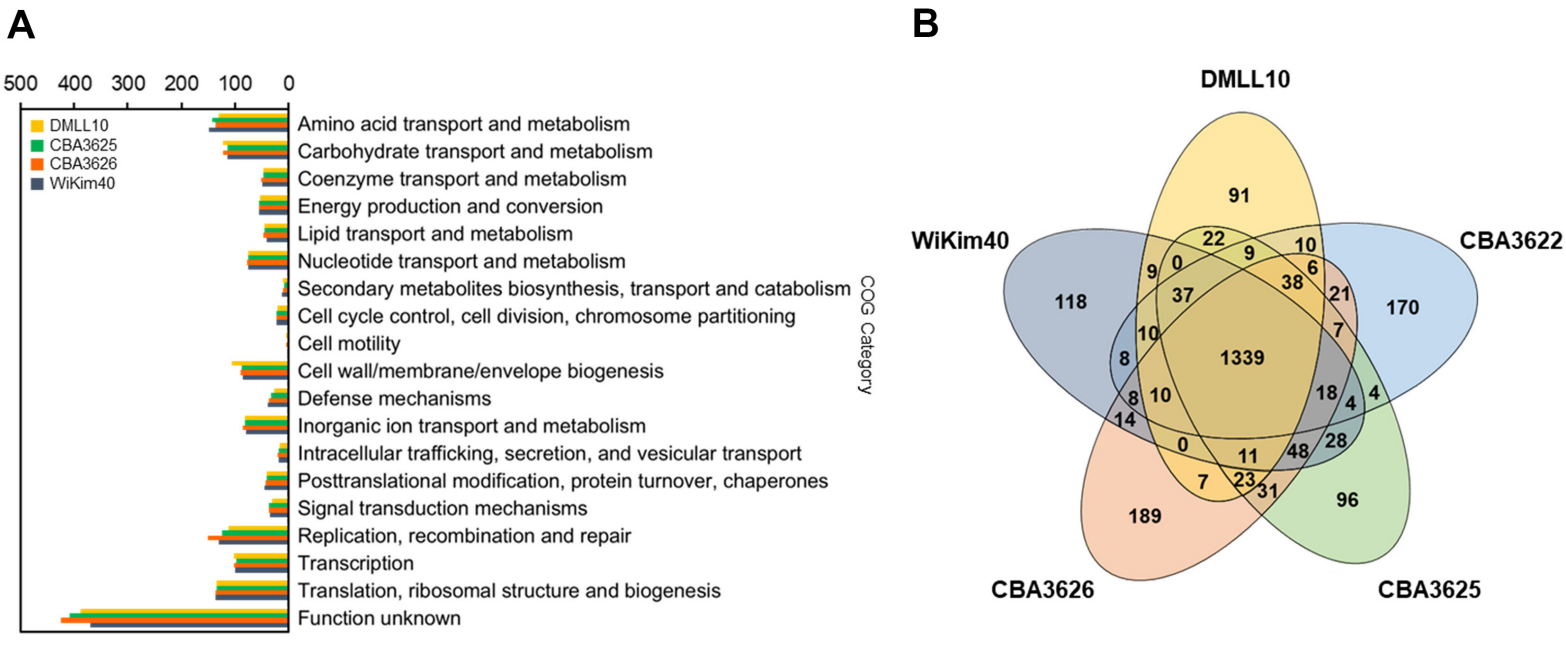
Comparative genomic analysis of *Leuconostoc lactis* DMLL10 with other strains. (**A**) COG functional categories of four strains, (**B**) Venn diagram showing the number of genes of orthologous CDSs (shared and unique ones) among the five strains.

The complete genome of strain DMLL10 contained a circular chromosome of 1,690,203 bp with a GC content of 43.4%. It did not possess a plasmid (Table 1). A total of 69 tRNA genes and 12 rRNA genes were identified in the genome of DMLL10. Genomic analysis predicted 1,646 Open Reading Frames (ORFs). Of them, 1,546 genes were functionally assigned to categories based on the COG database (Fig. 2A). The most abundant COG category was related to translation, ribosomal structure, and biogenesis (135 genes, 8.7%), followed by amino acid transport and metabolism (131 genes, 8.5%) and carbohydrate transport and metabolism (123 genes, 8.0%).

### Comparative Analysis of *Leuconostoc lactis* Genomes

As of April 2023, there were 38 registered genomes for *Leu. lactis*, of which only four strains were registered as complete genomes. The type strain, *Leu. lactis* KCTC 3528^T^, was registered as contigs. Thus, whole-genome comparison was conducted with four strains registered as complete genomes (Fig. 2).

To compare functional classification of genomes, we tried to compare them with four strains (CBA3622, CBA3625, CBA3626, and WiKim40) registered with complete genomes. However, COG results for strain CBA3622 were compared with three strains because they could not be confirmed in the EZBioCloud (https://www.ezbiocloud.net/) server (Fig. 2A). Except for the category of ‘function unknown’, the following four categories showed an average of more than 10% genes assigned to COG: ‘amino acid transport and metabolism’, ‘translation, ribosomal structure and biogenesis’, ‘replication, recombination and repair’, and ‘carbohydrate transport and metabolism’. There were more than 135 genes involved in ‘translation, ribosomal structure and biogenesis’, accounting for 11.1-11.7% of the total. Genes involved in ‘amino acid transport and metabolism’ accounted for 11.2-12.4%. Although rankings varied slightly by strain, the trend of gene assignment could be seen to be similar.

Gene pools shared by genomes of five *Leu. lactis* strains (DMLL10, CBA3622, CBA3625, CBA3626, and WiKim40) are depicted in a Venn diagram (Fig. 2B). These five strains shared 1,339 CDSs in their core genome, corresponding to approximately 75.6%–82.6% of their ORFs. Genomes of strain DMLL10 and CBA3625 had the smallest proportion (5.6%) of unique CDSs that were absent from the four other *Leu. lactis* genomes. In contrast, proportions of unique CDSs in genomes of strains CBA3622, CBA3626, and WiKim40 were 10.0%, 10.7%, and 7.1%, respectively. The majority of singleton-specific genes encoded hypothetical proteins (Table S1).

### Safety Properties of Strain DMLL10

The European Union Food Safety Authority (EFSA) has introduced the Qualified Presumption of Safety (QPS) approach to check the safety of microorganisms throughout the food chain [31]. *Leu. lactis* has been reported to be nonpathogenic [13]. It has been on the QPS list of the EFSA since 2007 [13]. *Leu. lactis* is on the IDF list as a fermented species of dairy products [14-17]. The above results are sufficient to assume that *Leu. lactis* is safe species. Unfortunately, *Leu. lactis* is not on the Food Materials list of the Ministry of Food and Drug Safety, Korea as of December 2022. In order to use strain DMLL10 as a starter for *kimchi* fermentation or soybean fermentation in Korea, it is currently necessary to register it as a temporary food ingredient. To this end, data on the safety of this strain should be submitted. Thus, the presence or absence of acquired antibiotic resistance gene and hemolysis were determined.

#### Acquired Antibiotic Resistance of DMLL10

EFSA issued guidelines to identify acquired antibiotic resistance to microorganisms used for food/feed use [28]. According to guidelines, antibiotic resistance activities of DMLL10 were determined based on its Minimum Inhibitory Concentrations (MICs) against eight antibiotics. The DMLL10 strain did not exhibit resistance to ampicillin, chloramphenicol, clindamycin, erythromycin, gentamicin, kanamycin, streptomycin, or tetracycline (Table 2).

Antibiotic resistance gene was then analyzed to determine whether there was an acquired antibiotic resistance gene on the basis of its genome. Based on COG functional classification, although six putative antibiotic resistance genes for multidrug resistance were identified in the genome of *Leu. lactis* DMLL10, resistance genes specific to the eight antibiotics suggested by QPS were not found (Table 3). All putative antibiotic-resistant genes of DMLL10 were encoded in the chromosome, not the plasmid. They were also detected in genomes of five *Leu. lactis* strains, KCTC 3528^T^, CBA3622, CBA3625, CBA3626, and WiKim40. In addition, those putative antibiotic resistance genes did not encode for horizontal transition factors such as plasmid. Instead, they were located in the chromosome. These results suggest that those putative antibiotic resistance gene should be intrinsic, not acquired. Indeed, functions of the six putative antibiotic resistance genes were annotated as efflux pumps or transporters, not resistance against specific antibiotics. Thus, we assumed that those genes belonged to the category of ‘reduction of antibiotic penetration and extrusion of antibiotics’, not ‘modification of antibiotics’, ‘inhibition of antibiotic and target binding’, and ‘modification of the binding site’ if those genes are involved in antibiotic resistance [32]. Nevertheless, this suggestion requires experimental proof. Therefore, those annotated antibiotic resistance genes in the DMLL10 genome encoded chromosomally might not contribute to antibiotic resistance. Consequently, the DMLL10 strain did not exhibit resistance to eight antibiotics. Acquired antibiotic resistance gene was not found.

#### Hemolysin and Enterotoxin in DMLL10

**Fig. 3:**
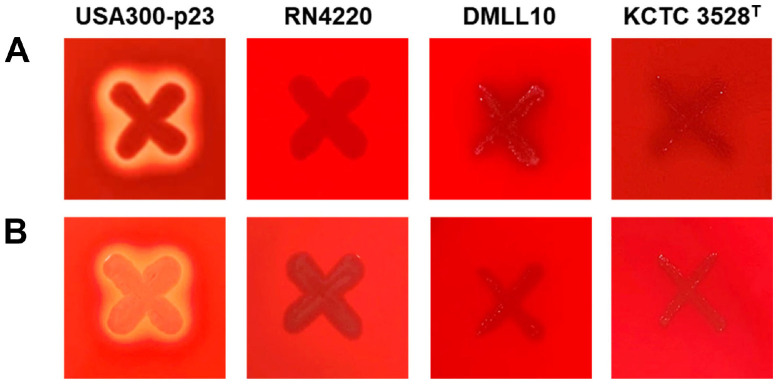
(A) α-Hemolytic activity and (B) β-hemolytic activity of *Leuconostoc lactis* DMLL10. *Staphylococcus aureus* strain USA300-p23 and RN4220 were used as positive and negative controls, respectively.

There are no guidelines for identifying toxin factors for *Leu. lactis*. However, according to guidelines of FAO/WHO for probiotics in food [33], toxin factors between the same species could be identified. *Leu. lactis* has already been on the QPS list since 2007 when the QPS system was introduced and *Leu. lactis* has been reported to be nonpathogenic [13]. Thus, this species is considered safe. In addition, *Leu. lactis* is listed in the IDF as a starter for dairy products, which indicates its safety [14-17]. In the present study, strain DMLL10 did not exhibit hemolysis on TSA media containing sheep and rabbit blood (Fig. 3). Hemolysis is a phenomenon caused by hemolysin. It is one of the representative endotoxins. Therefore, it can be seen that DMLL10 is a safe strain that does not show hemolysis. In order to verify this based on its genome, related genes were analyzed. Its related genes were not found as a result of checking them with keywords of 'hemolysin' and 'toxin' in the genome of DMLL10. Through this, we propose that DMLL10 strain is safe through phenotype and genomic analyses.

### Potential Role of Strain DMLL10 in Food Fermentation

#### Enzymatic Properties of Strain DMLL10

**Fig. 4:**
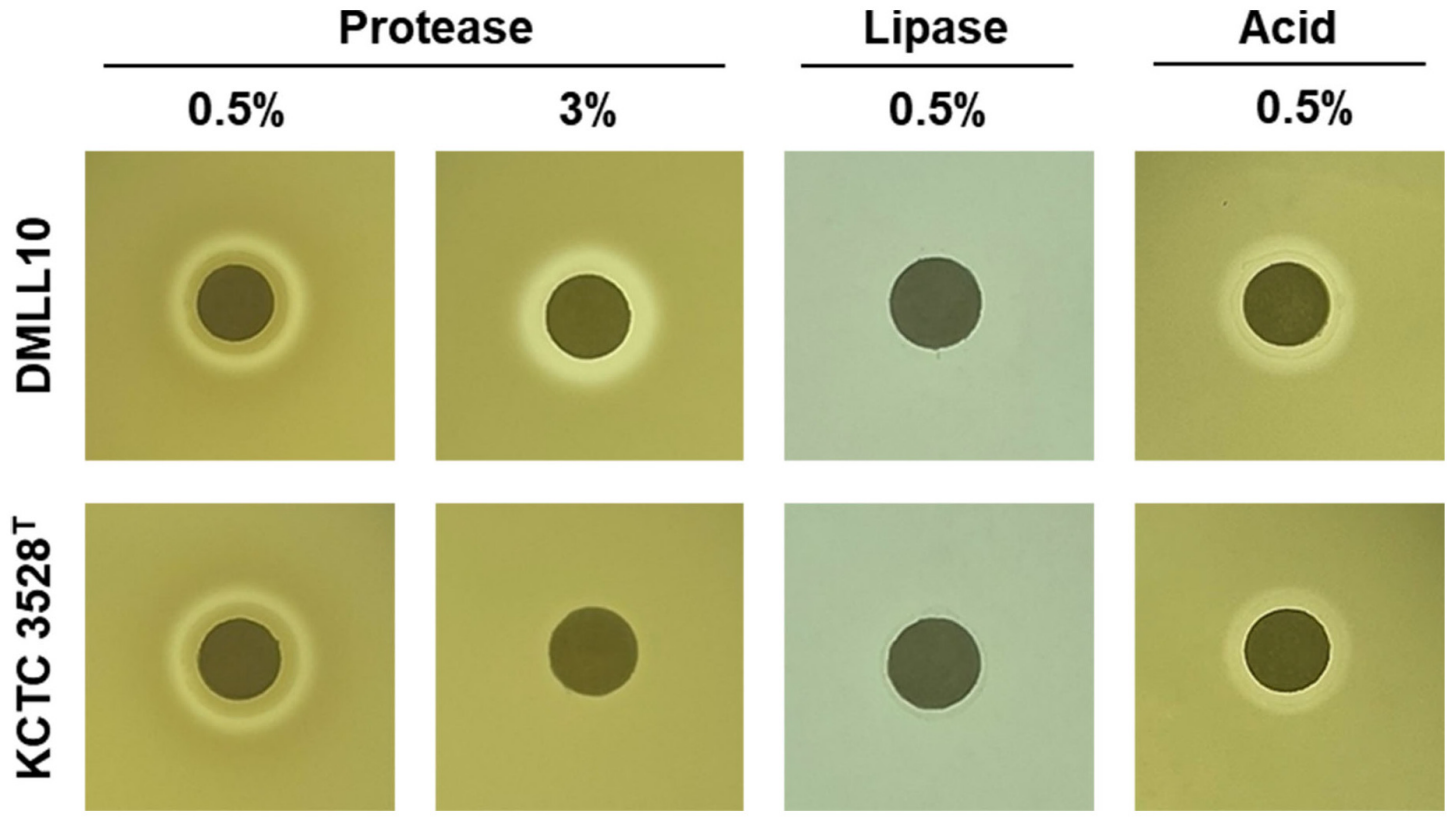
Enzymatic properties of *Leuconostoc lactis* DMLL10 on media. The formation of a clear zone around the filter paper disc is determined to be positive enzymatic activity.

*Leu. lactis* strain DMLL10 exhibited protease and acid production without showing lipase activities (Fig. 4 and Table S2). Especially, strain DMLL10 displayed a proteolytic activity on TSA supplemented with NaCl up to 3%(Fig. 4 and Table S2). Protease activity contribute to unique characteristics of fermented foods by breaking down proteins to organic acid, esters, amino acids, aldehydes, amines, and free fatty acids. These molecules can affect sensory properties during fermentation. Strain DMLL10 possessed 41 genes related proteolytic activity (Table 4). Comparative genomic analysis of *Leu. lactis* with five other strains revealed that the genome of strain DMLL10 possessed three more genes, serine hydrolase (PH197_05045, E.C. 3.4.16.4), prolyl oligopeptidase family serine peptidase (PH197_00225), and cysteine hydrolase (PH197_04140, E.C. 3.5.1.110), associated with proteolytic enzyme compared to five other strains (Table 4). It is necessary to confirm whether those genes could further affect protease activity when salt concentration is higher. These results imply that *Leu. lactis* strain DMLL10 might affect protein degradation under salt stress during fermentation.

#### Homo- and Hetero-Lactic Fermentative Pathway

**Fig. 5:**
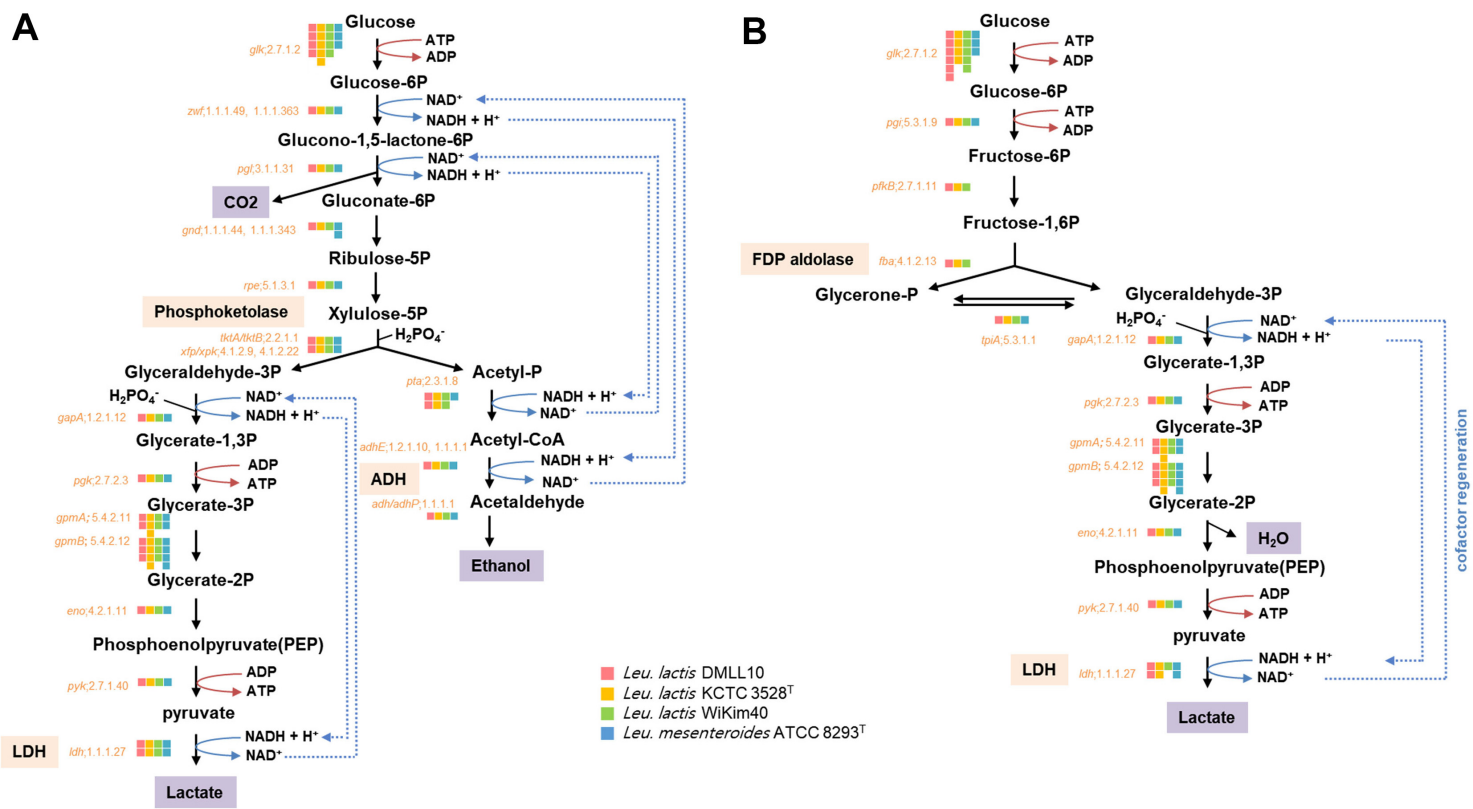
Predicted (A) hetero- and (B) homo- lactic fermentative pathways of three *Leuconostoc lactis* strains and *Leuconostoc mesenteroides*. Enzyme-encoding genes and E.C. number are displayed in orange. Metabolites are shown in light purple box. Key enzyme genes for fermentation are shown in light pink box. Gene possession was marked with a box of colors corresponding to each strain.

It is well known that *Leuconostoc* spp. are obligatory hetero-lactic fermentative bacteria [34]. *Leuconostoc lactis* strain DMLL10 possessed genes related to hetero-lactic fermentative pathway, which produced lactate, CO_2_, and ethanol from glucose (Fig. 5A). Interestingly, *Leu. lactis* strain DMLL10 also possessed genes related to the homo-lactic fermentative pathway, which produced two lactates from glucose (Fig. 5B). CO_2_ and ethanol produced through lactic acid fermentation give a refreshing feeling. Lactic acid lowers pH and inhibits the growth of spoilage and pathogenic microorganisms. In particular, hetero-lactic fermentation can lower pH slowly because the production of lactic acid is lower than homo-lactic fermentation. This can delay souring of fermented foods and extend the edible period. Also, hetero-lactic fermentation gives a sense of coolness or refreshing peeling due to CO_2_ and ethanol. It is not possible to determine which of these two ferments is appropriate. However, fermentation periods for sauerkraut and *kimchi* are mainly considered propriate for eating when hetero-lactic fermentative LAB dominate. In the case of dairy products, homo-lactic fermentative LAB are mainly involved because the storage period needs to extend by forming a low pH. Based on the genome, strain DMLL10 has all genes for homo and hetero-lactic fermentation, while *Leu. mesenteroides* is a hetero-lactic fermentative LAB because there is no FDP aldolase gene (E.C. 4.1.2.13) (Fig. 5B). So far, there has been a lack of research on whether *Leu. lactis* proceeds with both fermentations. If it does, whether they proceed simultaneously and whether fermentation varies depending on conditions are unknown. Thus, more related research is needed. However, it is considered effective if these two fermentations can be adjusted according to the purpose.

#### Antimicrobial Activities of Strain DMLL10

**Fig. 6:**
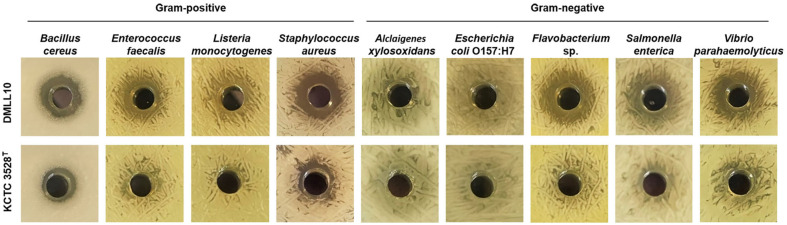
Antibacterial activities of strain DMLL10 against food pathogens.

*Leuconostoc lactis* strain DMLL10 showed antagonistic activities to inhibit pathogenic and/or spoilage microorganism in food on agar well diffusion method against seven food pathogens (*Bacillus cereus*, *Enterococcus faecalis*, *Flavobacterium* sp., *Listeria monocytogenes*, *Staphylococcus aureus*, *Salmonella enterica*, and *Vibrio parahaemolyticus*). However, it showed weak activities in inhibiting the growth of *Alcaligenes xylosoxidans* and *Escherichia coli* O157:H7 (Fig. 6 and Table S3). Compared with KCTC 3528^T^, the type strain, strain DMLL10 showed stronger antibacterial activity. The bacteriocin gene was searched for a genome-based explanation of its anti-microbial activity. However, no gene related to bacteriocin was identified except for a lactococcin-A immunity protein (PH197_04005) gene. Therefore, more research is needed on genes that contribute to its antibacterial activity.

## Conclusion

Safety and technological properties of *Leu. lactis* DMLL10 derived from *Jeonbok-baechu-kimchi* as a starter candidate for food fermentation were determined. In addition, genomic analysis was performed to determine its safety and technological activities. Strain DMLL10 showed sensitivity to antibiotics and do not show hemolysis. Its phenotypic activities were confirmed through genomic analysis. The proteolytic activity of DMLL10 strain is expected to contribute to the production of amino acids by decomposing proteins in fermented foods, thereby improving functional properties of fermented foods. In addition, its antibacterial activity will contribute to the safety of fermented foods by inhibiting the growth of spoilage bacteria or food poisoning bacteria present in the raw material or environment during the fermentation period. Until now, most bacteria used for *kimchi* fermentation have been *Leu. mesenteroides*. *Leu. lactis* DMLL10 derived through this experiment is the same genus as *Leu. mesenteroides*. It showed enzymatic and antibacterial activities. Thus, it has potential as a novel starter candidate. It is believed that it will contribute to the production of fermented foods as a fermented species along with *Leu. mesenteroides*. However, more research is needed on commonalities and differences between these two species for producing fermented foods in the future.

## Supplemental Materials

Supplementary data for this paper are available on-line only at http://jmb.or.kr.



## Figures and Tables

**Table 1:** General genomic and specific phenotypic features of six *Leuconostoc lactis* strains.

Feature	DMLL10	KCTC 3528^T^	CBA3622	CBA3625	CBA3626	WiKim40
Size (bp)	1,690,203	2,011,205	1,787,635	1,791,608	1,839,813	1,788,069
Chromosome size (bp)	1,690,203	-	1,635,644	1,781,455	1,790,249	1,737,502
Plasmid 1	-	-	46,945	10,153	49,564	20,388
Plasmid 2	-	-	28,768	-	-	19,726
Plasmid 3	-	-	76,278	-	-	10,453
G+C content (mol%)	43.41	42.60	42.92	43.35	43.15	43.11
No. of plasmids	0	-	3	1	1	3
Open reading frames	1,646	-	1,811	1,816	1,915	1,767
CDSs assigned by COG ^c^	1,546	-	-	1,577	1,648	1,559
No. of rRNAs	12	-	12	12	12	12
No. of tRNAs	69	-	68	67	72	68
Other RNA	-	-	3	3	3	3
Contigs	1	1,151	4	2	2	4
Origin	*Kimchi*	Milk	*Kimchi*	*Kimchi*	*Kimchi*	*Kimchi*
Accession No.	CP116456	AEOR01000001-AEOR01001151	CP042420-CP042423	CP042387-CP042388	CP042390-CP042391	CP016598-CP016601
References	This study	(Type strain)	[35]	[35]	[35]	[35]

Abbreviations: CDS, coding DNA sequence; COG, Clusters of Orthologous Group of proteins; ^T^, Type strain; -, unknown.

**Table 2:** Minimal inhibitory concentrations of *Leuconostoc lactis* DMLL10 against eight antibiotics.

Antibiotics	MIC (mg/l)	Breakpoint*
Ampicillin	1	2
Chloramphenicol	4	4
Clindamycin	0.5	1
Erythromycin	0.5	1
Gentamicin	0.5	16
Kanamycin	0.5	16
Streptomycin	0.5	64
Tetracycline	0.5	8

*EFSA Breakpoint for *Leuconostoc* sp.

**Table 3:** Annotated antibiotic resistance determinants identified in the DMLL10 genome and five other *Leuconostoc lactis* strains.

DMLL10	Product	KEGG	COG	Presence of gene in *Leu. lactis* genomes				
				KCTC 3528T	CBA3622	CBA3625	CBA3626	WiKim40
PH197_00085	Multidrug resistance efflux transporter family protein		S	●	●	●	-	●
PH197_01685	MFS transporter	K03446	G	●	●	●	●	●
PH197_05335	Multidrug efflux MFS transporter	K08161	G	●	●	●	●	●
PH197_06315	Multidrug efflux SMR transporter	K03297	P	●	●	●	●	●
PH197_06765	MFS transporter	K08153	G	●	●	●	●	●
PH197_08040	MDR family MFS transporter	K18926	G	●	●	●	●	●

^T^, Type strain; ●, identified; -, Not identified; KEEG, The Kyoto Encyclopedia of Genes and Genomes; COG, Clusters of Orthologous Group of proteins.

**Table 4:** Annotated protease genes identified in the DMLL10 genome and five other *Leuconostoc lactis* strains.

Category	DMLL10 Gene locus	Product	E.C. No.	KEGG	COG	Presence of gene in *Leu. lactis* genomes				
						KCTC 3528^T^	CBA3622	CBA3625	CBA3626	WiKim40
Protease										
	PH197_01785	ATP-dependent Clp protease proteolytic subunit	3.4.21.92	K01358	O	●	●	●	●	●
	PH197_02805	Zinc metalloprotease HtpX	3.4.24.-	K03799	O	●	●	●	●	●
	PH197_03005	RIP metalloprotease RseP	3.4.24.-	K11749	M	●	●	●	●	-
	PH197_06795	ATP-dependent zinc metalloprotease FtsH	3.4.24.-	K03798	O	●	●	●	●	●
	PH197_00590	Endopeptidase	3.4.24.-	K07386	O	●	●	●	●	●
	PH197_00785	Trypsin-like peptidase domain-containing protein	3.4.21.107	K04771	O	●	●	●	●	●
	PH197_01305	M3 family oligoendopeptidase	3.4.24.-	K08602	E	●	●	●	●	●
	PH197_01600	Oligoendopeptidase F	3.4.24.-	K08602	E	●	●	●	●	●
	PH197_02105	Type II CAAX endopeptidase family protein	-	K07052	S	●	●	●	●	●
	PH197_02110	Xaa-Pro peptidase family protein	3.4.13.9	K01271	E	●	●	●	●	●
	PH197_02260	Glutamyl aminopeptidase	3.4.11.7	K01261	E	●	●	●	●	●
	PH197_02305	M15 family metallopeptidase	3.4.17.14	K07260	M	-	●	-	-	●
	PH197_02310	Sapep family Mn(2+)-dependent dipeptidase	3.5.1.18	K01439	E	-	●	-	-	●
	PH197_02375	Dipeptidase PepV	3.4.13.-	K01274	E	●	●	●	●	●
	PH197_03230	Carboxypeptidase M32	3.4.17.19	K01299	E	●	●	●	●	●
	PH197_03505	M1 family metallopeptidase	3.4.11.2	K01256	E	●	●	●	●	●
	PH197_04045	C39 family peptidase	-	K21125	S	●	●	●	●	-
	PH197_04105	Type II CAAX endopeptidase family protein	-	K07052	S	-	●	●	-	●
	PH197_04400	Peptidase T	3.4.11.4	K01258	E	●	●	●	●	●
	PH197_04840	M24 family metallopeptidase	3.4.11.9	K01262	E	●	●	●	●	●
	PH197_05490	Type I methionyl aminopeptidase	3.4.11.18	K01265	J	●	●	●	●	●
	PH197_07055	Trypsin-like peptidase domain-containing protein	3.4.21.107	K04771	O	●	●	●	●	●
	PH197_07095	Aminopeptidase	3.4.11.-	K19689	E	●	●	●	●	●
	PH197_07515	ImmA/IrrE family metallo-endopeptidase	-	-	-	●	-	-	●	-
Serine hydrolase										
	PH197_00225	Prolyl oligopeptidase family serine peptidase	-	-	I	-	-	-	-	-
	PH197_00380	Serine hydrolase	-	-	S	-	●	●	●	●
	PH197_01455	Serine hydrolase	-	-	S	●	●	●	●	●
	PH197_01670	Serine hydrolase	3.1.1.103	K22580	V	●	●	●	●	●
	PH197_02285	SepM family pheromone-processing serine protease	-	K07177	T	●	●	●	●	●
	PH197_02300	Serine hydrolase	3.4.16.4	K07258	M	-	●	-	-	●
	PH197_02320	Class A beta-lactamase-related serine hydrolase	3.5.2.6	K17836	V	-	●	-	-	●
	PH197_05045	Serine hydrolase	3.4.16.4	K01286	V	-	-	-	-	-
	PH197_05505	Rhomboid family intramembrane serine protease	3.4.21.105	K19225	S	●	●	●	●	●
Cysteine hydrolase										
	PH197_04140	Cysteine hydrolase	3.5.1.110	K09020	Q	-	-	-	-	-
	PH197_06155	YiiX/YebB-like N1pC/P60 family cysteine hydrolase	-	-	S	●	●	●	●	●
Others										
	PH197_01050	LysM peptidoglycan-binding domaincontaining protein/Lysin motif domain	3.4.-.-	K21471	M	●	●	●	●	●
	PH197_01055	NlpC/P60 family protein/endopeptidase domain like	3.4.-.-	K21471	M	●	●	●	●	●
	PH197_01060	LysM peptidoglycan-binding domaincontaining protein/Lysin motif domain	3.4.-.-	K19224	S	●	●	●	●	●
	PH197_05210	Peptide deformylase/bacteria to generate the mature free N-terminal polypeptide and formate	3.5.1.88	K01462	J	●	●	●	●	●
	PH197_06100	Pitrilysin family protein/Insulysin	3.4.24.56	K01408	O	●	●	●	●	●
	PH197_06105	Pitrilysin family protein/Probable inactive metalloprotease YmfF	3.4.24.-	K07263	O	●	●	●	●	●

^T^, Type strain; ●, identified; -, Not identified; E.C. No., European Community number; KEEG, The Kyoto Encyclopedia of Genes and Genomes; COG, Clusters of Orthologous Group of protein.
