# Supplementary material for: Novel Strain Leuconostoc lactis DMLL10 from Traditional Korean Fermented Kimchi as a Starter Candidate for Fermented Foods
Source: J Microbiol Biotechnol. 2023 Sep 7;33(12):1625–34. doi: 10.4014/jmb.2306.06056 (PMC10772556; doi:10.4014/jmb.2306.06056)
Supplement: Supplementary file 1 [file jmb-33-12-1625-supple.pdf]

J. Microbiol. Biotechnol.
https://doi.org/10.4014/jmb.2306.06056

Supplementary Tables

Table S1. List of singletons generated by comparing genomes of five *Leuconostoc lactis* strains.

Strain	Gene locus	Product
DMLL10	PH197_00225	Prolyl oligopeptidase family serine peptidase
	PH197_00310	KilA-N domain-containing protein
	PH197_00315	Hypothetical protein
	PH197_00335	Serine/threonine protein kinase
	PH197_01465	Hypothetical protein
	PH197_02040	IS3 family transposase
	PH197_02290	Hypothetical protein
	PH197_02645	DUF916 domain-containing protein
	PH197_03195	Hypothetical protein
	PH197_03715	NAD(P)-binding domain-containing protein
	PH197_03865	Cold-shock protein
	PH197_03960	Hypothetical protein
	PH197_03965	GNAT family N-acetyltransferase
	PH197_04140	Cysteine hydrolase
	PH197_04145	Hypothetical protein
	PH197_04150	DUF1440 domain-containing protein
	PH197_04155	SDR family NAD(P)-dependent oxidoreductase
	PH197_04160	Hypothetical protein
	PH197_04165	Putative bacteriocin export ABC transporter
	PH197_04175	Hypothetical protein
	PH197_04180	Cation-translocating P-type ATPase
	PH197_04185	KxYKxGKxW signal peptide domain-containing protein
	PH197_04190	Peptidoglycan amidohydrolase family protein
	PH197_04195	DUF1430 domain-containing protein
	PH197_04200	SMR family transporter
	PH197_04255	Hypothetical protein
	PH197_04790	Hypothetical protein
	PH197_04880	DTDP-4-dehydrorhamnose reductase
	PH197_04885	DTDP-glucose 4,6-dehydratase
	PH197_04895	Glucose-1-phosphate thymidylyltransferase RfbA
	PH197_04900	Hypothetical protein
	PH197_04905	Hypothetical protein
	PH197_04910	Glycosyltransferase
	PH197_04915	Polysaccharide biosynthesis C-terminal domain-containing protein
	PH197_04930	Oligosaccharide flippase family protein
	PH197_04935	Nucleotide sugar dehydrogenase
	PH197_04940	Transposase
	PH197_04945	IS3 family transposase
	PH197_04950	Wzz/FepE/Etk N-terminal domain-containing protein
	PH197_04955	IS1380 family transposase
	PH197_04960	LCP family protein
	PH197_04965	ISL3 family transposase
	PH197_04970	Hypothetical protein
	PH197_04975	Glycosyltransferase family 4 protein
	PH197_04990	Nucleotide sugar dehydrogenase
	PH197_04995	NAD-dependent epimerase/dehydratase family protein
	PH197_05000	Hypothetical protein

	PH197_05005	Glycosyltransferase
	PH197_05010	Glycosyltransferase
	PH197_05015	Glycosyltransferase family 4 protein
	PH197_05045	Serine hydrolase
	PH197_05055	YdcF family protein
	PH197_05070	Arsenate reductase (thioredoxin)
	PH197_05075	Cupin domain-containing protein
	PH197_05080	VIT family protein
	PH197_05085	VIT family protein
	PH197_05090	Cation:proton antiporter
	PH197_05095	Chloride channel protein
	PH197_05100	Transposase
	PH197_05105	DDE-type integrase/transposase/recombinase
	PH197_05170	Hypothetical protein
	PH197_05180	Hypothetical protein
	PH197_05380	ROK family protein
	PH197_05665	Hypothetical protein
	PH197_05670	Molecular chaperone Tir
	PH197_05675	SIR2 family protein
	PH197_05745	Hypothetical protein
	PH197_05755	Transposase
	PH197_05760	DTDP-glucose 4,6-dehydratase
	PH197_05765	Glucose-1-phosphate thymidylyltransferase RfbA
	PH197_05775	Hypothetical protein
	PH197_05785	Glycosyltransferase family 2 protein
	PH197_05790	NAD-dependent epimerase/dehydratase family protein
	PH197_05795	IspD/TarI family cytidylyltransferase
	PH197_05800	LicD family protein
	PH197_05805	Glycosyltransferase family 2 protein
	PH197_05810	Glycosyltransferase family 4 protein
	PH197_07260	Hypothetical protein
	PH197_07265	Hypothetical protein
	PH197_07275	Hypothetical protein
	PH197_07280	Hypothetical protein
	PH197_07390	Hypothetical protein
	PH197_07395	Hypothetical protein
	PH197_07400	Hypothetical protein
	PH197_07405	Hypothetical protein
	PH197_07490	DUF2188 domain-containing protein
	PH197_07495	Hypothetical protein
	PH197_07520	DUF4352 domain-containing protein
	PH197_07665	AAA family ATPase
	PH197_07670	DUF6414 family protein
	PH197_08305	Hypothetical protein
CBA3622	FGL80_RS00345	DTDP-4-dehydrorhamnose reductase
	FGL80_RS00355	DTDP-4-dehydrorhamnose 3,5-epimerase family protein
	FGL80_RS00360	Glucose-1-phosphate thymidylyltransferase RfbA
	FGL80_RS00365	Hypothetical protein
	FGL80_RS00370	Glycosyltransferase family 2 protein
	FGL80_RS00375	Glycosyltransferase
	FGL80_RS00380	Hypothetical protein
	FGL80_RS00395	Glycosyltransferase
	FGL80_RS00400	LicD family protein
	FGL80_RS00405	Acyltransferase
	FGL80_RS00410	Capsular polysaccharide synthesis protein
	FGL80_RS00415	Beta-1,6-N-acetylglucosaminyltransferase
	FGL80_RS01340	MFS transporter

FGL80_RS01980	Hypothetical protein
FGL80_RS01985	Hypothetical protein
FGL80_RS03130	Hypothetical protein
FGL80_RS03255	ROK family protein
FGL80_RS06630	Cold-shock protein
FGL80_RS06720	Hypothetical protein
FGL80_RS06760	DUF2785 domain-containing protein
FGL80_RS06880	Transposase
FGL80_RS06885	IS3 family transposase
FGL80_RS06900	Hypothetical protein
FGL80_RS06905	Hypothetical protein
FGL80_RS06915	AbiH family protein
FGL80_RS06985	D-2-hydroxyacid dehydrogenase
FGL80_RS07070	DTDP-glucose 4,6-dehydratase
FGL80_RS07660	Transposase
FGL80_RS07665	IS3 family transposase
FGL80_RS07670	Hypothetical protein
FGL80_RS07675	Fic family protein
FGL80_RS07695	SGNH/GDSL hydrolase family protein
FGL80_RS07700	Hypothetical protein
FGL80_RS07705	Hypothetical protein
FGL80_RS07710	Hypothetical protein
FGL80_RS07715	IS3 family transposase
FGL80_RS07720	Transposase
FGL80_RS07725	Hypothetical protein
FGL80_RS07745	DUF4145 domain-containing protein
FGL80_RS07830	Hypothetical protein
FGL80_RS07840	Helix-turn-helix transcriptional regulator
FGL80_RS07845	XRE family transcriptional regulator
FGL80_RS07850	Hypothetical protein
FGL80_RS07865	Cold-shock protein
FGL80_RS07925	Hypothetical protein
FGL80_RS07945	Hypothetical protein
FGL80_RS07960	Hypothetical protein
FGL80_RS07965	Hypothetical protein
FGL80_RS07970	DUF4868 domain-containing protein
FGL80_RS07975	Transposase
FGL80_RS07980	DDE-type integrase/transposase/recombinase
FGL80_RS07985	Hypothetical protein
FGL80_RS08195	Hypothetical protein
FGL80_RS08200	Replication initiation factor domain-containing protein
FGL80_RS08205	Hypothetical protein
FGL80_RS08210	Conjugal transfer protein
FGL80_RS08215	TcpD family membrane protein
FGL80_RS08220	TcpE family conjugal transfer membrane protein
FGL80_RS08225	Serine-rich aggregation substance UasX
FGL80_RS08230	Antirestriction protein ArdA
FGL80_RS08235	Hypothetical protein
FGL80_RS08240	ATP-binding protein
FGL80_RS08250	Peptide ABC transporter permease
FGL80_RS08255	Thioredoxin family protein
FGL80_RS08260	Hypothetical protein
FGL80_RS08265	Hypothetical protein
FGL80_RS08270	Hypothetical protein
FGL80_RS08280	Hypothetical protein
FGL80_RS08285	Type II toxin-antitoxin system RelB/DinJ family antitoxin
FGL80_RS08290	Type II toxin-antitoxin system PemK/MazF family toxin

FGL80_RS08305	Hypothetical protein
FGL80_RS08310	Lrp/AsnC family transcriptional regulator
FGL80_RS08315	ATP-binding cassette domain-containing protein
FGL80_RS08320	Thioredoxin
FGL80_RS08325	FAD-dependent oxidoreductase
FGL80_RS08330	Metal-sensitive transcriptional regulator
FGL80_RS08335	MBL fold metallo-hydrolase
FGL80_RS08340	Translation elongation factor 4
FGL80_RS08350	NAD(P)H-binding protein
FGL80_RS08360	Recombinase family protein
FGL80_RS08365	Integrase core domain-containing protein
FGL80_RS08385	Hypothetical protein
FGL80_RS08390	Heavy metal translocating P-type ATPase
FGL80_RS08395	MerR family DNA-binding transcriptional regulator
FGL80_RS08410	MerR family transcriptional regulator
FGL80_RS08415	IS3 family transposase
FGL80_RS08420	Transposase
FGL80_RS08445	DUF6414 family protein
FGL80_RS08450	AAA family ATPase
FGL80_RS08465	Hypothetical protein
FGL80_RS08470	Hypothetical protein
FGL80_RS08475	ATPase, T2SS/T4P/T4SS family
FGL80_RS08540	DUF1345 domain-containing protein
FGL80_RS08550	Aldo/keto reductase
FGL80_RS08600	DNA methyltransferase
FGL80_RS08605	DEAD/DEAH box helicase family protein
FGL80_RS08610	Hypothetical protein
FGL80_RS08615	3-hydroxyacyl-CoA dehydrogenase
FGL80_RS08630	AAA family ATPase
FGL80_RS08665	MobV family relaxase
FGL80_RS08680	Heavy-metal-associated domain-containing protein
FGL80_RS08685	DNA starvation/stationary phase protection protein
FGL80_RS08690	Crp/Fnr family transcriptional regulator
FGL80_RS08715	Hypothetical protein
FGL80_RS08720	Helix-turn-helix transcriptional regulator
FGL80_RS08725	Hypothetical protein
FGL80_RS08735	RepB family plasmid replication initiator protein
FGL80_RS08740	MobV family relaxase
FGL80_RS08745	Helix-turn-helix transcriptional regulator
FGL80_RS08755	Heavy-metal-associated domain-containing protein
FGL80_RS08765	SDR family oxidoreductase
FGL80_RS08780	GlsB/YeaQ/YmgE family stress response membrane protein
FGL80_RS08785	Alkaline shock response membrane anchor protein AmaP
FGL80_RS08790	DUF2273 domain-containing protein
FGL80_RS08795	Asp23/Gls24 family envelope stress response protein
FGL80_RS08800	DUF1345 domain-containing protein
FGL80_RS08805	GlsB/YeaQ/YmgE family stress response membrane protein
FGL80_RS08810	Aldo/keto reductase
FGL80_RS08815	Sugar porter family MFS transporter
FGL80_RS08820	Polysaccharide pyruvyl transferase family protein
FGL80_RS08830	Multicopper oxidase domain-containing protein
FGL80_RS08840	Hypothetical protein
FGL80_RS08845	MobV family relaxase
FGL80_RS08850	RepB family plasmid replication initiator protein
FGL80_RS08855	HTH domain-containing protein
FGL80_RS08860	DNA methyltransferase
FGL80_RS08865	DEAD/DEAH box helicase family protein

	FGL80_RS08870	Hypothetical protein
	FGL80_RS08875	3-hydroxyacyl-CoA dehydrogenase
	FGL80_RS08880	Oleate hydratase
	FGL80_RS08885	Transposase
	FGL80_RS08890	AAA family ATPase
	FGL80_RS08925	MobV family relaxase
	FGL80_RS08930	Helix-turn-helix transcriptional regulator
	FGL80_RS08940	Heavy-metal-associated domain-containing protein
	FGL80_RS08945	DNA starvation/stationary phase protection protein
	FGL80_RS08950	Crp/Fnr family transcriptional regulator
	FGL80_RS08960	Magnesium transporter CorA family protein
	FGL80_RS08970	Site-specific integrase
	FGL80_RS08975	Hypothetical protein
	FGL80_RS08980	Helix-turn-helix transcriptional regulator
	FGL80_RS08985	Hypothetical protein
	FGL80_RS08990	Helix-turn-helix domain-containing protein
	FGL80_RS08995	RepB family plasmid replication initiator protein
	FGL80_RS09000	MobV family relaxase
	FGL80_RS09005	Helix-turn-helix transcriptional regulator
	FGL80_RS09015	Heavy-metal-associated domain-containing protein
	FGL80_RS09055	Hypothetical protein
	FGL80_RS09060	Hypothetical protein
	FGL80_RS09065	Hypothetical protein
	FGL80_RS09080	Hypothetical protein
	FGL80_RS09085	Hypothetical protein
	FGL80_RS09090	Hypothetical protein
	FGL80_RS09095	Hypothetical protein
	FGL80_RS09100	Hypothetical protein
	FGL80_RS09110	MFS transporter
	FGL80_RS09115	Hypothetical protein
	FGL80_RS09135	Hypothetical protein
	FGL80_RS09140	Hypothetical protein
	FGL80_RS09145	DEAD/DEAH box helicase family protein
	FGL80_RS09150	Hypothetical protein
	FGL80_RS09155	Hypothetical protein
	FGL80_RS09165	Hypothetical protein
	FGL80_RS09175	HAD-IC family P-type ATPase
	FGL80_RS09185	Hypothetical protein
	FGL80_RS09190	DEAD/DEAH box helicase family protein
	FGL80_RS09195	Hypothetical protein
	FGL80_RS09200	Hypothetical protein
	FGL80_RS09210	Hypothetical protein
	FGL80_RS09220	HAD-IC family P-type ATPase
CBA3625	FGL83_RS00355	Hypothetical protein
	FGL83_RS00360	Peptidoglycan recognition family protein
	FGL83_RS00365	Phage holin family protein
	FGL83_RS00460	Hypothetical protein
	FGL83_RS00465	Hypothetical protein
	FGL83_RS00475	Hypothetical protein
	FGL83_RS00480	DUF722 domain-containing protein
	FGL83_RS00485	Hypothetical protein
	FGL83_RS00505	DTDP-glucose pyrophosphorylase
	FGL83_RS00515	Single-stranded DNA-binding protein
	FGL83_RS00535	DNA replication protein
	FGL83_RS00575	Hypothetical protein
	FGL83_RS00580	Hypothetical protein
	FGL83_RS01740	ROK family protein

FGL83_RS02875	Hypothetical protein
FGL83_RS03055	DNA topology modulation protein
FGL83_RS03060	Class I SAM-dependent methyltransferase
FGL83_RS03065	GIY-YIG nuclease family protein
FGL83_RS03170	Hypothetical protein
FGL83_RS04295	CRISPR-associated endonuclease Cas2
FGL83_RS04300	Type II-A CRISPR-associated protein Csn2
FGL83_RS04605	Hypothetical protein
FGL83_RS04935	Hypothetical protein
FGL83_RS05345	Hypothetical protein
FGL83_RS05360	Hypothetical protein
FGL83_RS05365	Hypothetical protein
FGL83_RS05370	Transcriptional regulator
FGL83_RS05375	Hypothetical protein
FGL83_RS05380	Phage antirepressor KilAC domain-containing protein
FGL83_RS05390	Hypothetical protein
FGL83_RS05405	Hypothetical protein
FGL83_RS05420	Hypothetical protein
FGL83_RS05425	DUF1351 domain-containing protein
FGL83_RS05430	ERF family protein
FGL83_RS05440	Putative HNHc nuclease
FGL83_RS05450	Helix-turn-helix transcriptional regulator
FGL83_RS05465	DUF3850 domain-containing protein
FGL83_RS05485	Hypothetical protein
FGL83_RS05495	Hypothetical protein
FGL83_RS05510	DUF1056 family protein
FGL83_RS05520	Clp protease ClpP
FGL83_RS05525	Phage major capsid protein
FGL83_RS05530	Head-tail connector protein
FGL83_RS05540	HK97 gp10 family phage protein
FGL83_RS05545	DUF806 family protein
FGL83_RS05550	Phage tail protein
FGL83_RS05555	Phage tail tube assembly chaperone
FGL83_RS05560	Tape measure protein
FGL83_RS05565	Transglycosylase SLT domain-containing protein
FGL83_RS05570	Phage tail family protein
FGL83_RS05575	Prophage endopeptidase tail family protein
FGL83_RS05580	Hypothetical protein
FGL83_RS05585	GDSL-type esterase/lipase family protein
FGL83_RS05600	Hypothetical protein
FGL83_RS05605	Hypothetical protein
FGL83_RS05610	Hypothetical protein
FGL83_RS05615	Hypothetical protein
FGL83_RS06265	Glycosyltransferase family 4 protein
FGL83_RS06270	Hypothetical protein
FGL83_RS06275	O-antigen polysaccharide polymerase Wzy
FGL83_RS06280	Hypothetical protein
FGL83_RS06285	Acyltransferase
FGL83_RS06290	Beta-1,6-N-acetylglucosaminyltransferase
FGL83_RS06850	Hypothetical protein
FGL83_RS07275	IS3 family transposase
FGL83_RS07580	Hypothetical protein
FGL83_RS07775	Hypothetical protein
FGL83_RS07865	IS6 family transposase
FGL83_RS07870	FAD-dependent oxidoreductase
FGL83_RS07875	Hypothetical protein
FGL83_RS07880	IS6 family transposase

	FGL83_RS07885	Plasmid pRiA4b ORF-3 family protein
	FGL83_RS07895	Hypothetical protein
	FGL83_RS07915	IS6 family transposase
	FGL83_RS07920	Hypothetical protein
	FGL83_RS08005	DUF389 domain-containing protein
	FGL83_RS08010	Potassium transporter TrkG
	FGL83_RS08185	ABC transporter transmembrane domain-containing protein
	FGL83_RS08190	Hypothetical protein
	FGL83_RS08195	ATP-binding cassette domain-containing protein
	FGL83_RS08200	Hypothetical protein
	FGL83_RS08205	Hypothetical protein
	FGL83_RS08210	Hypothetical protein
	FGL83_RS08705	Histidine phosphatase family protein
	FGL83_RS09040	Helix-turn-helix transcriptional regulator
	FGL83_RS09065	AraC family transcriptional regulator
	FGL83_RS09070	GNAT family N-acetyltransferase
	FGL83_RS09110	Hypothetical protein
	FGL83_RS09120	Hypothetical protein
	FGL83_RS09140	Hypothetical protein
	FGL83_RS09155	Hypothetical protein
	FGL83_RS09165	Hypothetical protein
	FGL83_RS09170	Hypothetical protein
	FGL83_RS09180	Hypothetical protein
	FGL83_RS09190	Cas9 endonuclease PAM-interacting domain-containing protein
	FGL83_RS09195	Hypothetical protein
CBA3626	FGL78_RS00170	Peptidoglycan recognition family protein
	FGL78_RS00175	Hypothetical protein
	FGL78_RS00180	Hypothetical protein
	FGL78_RS00190	Hypothetical protein
	FGL78_RS00195	Phage tail protein
	FGL78_RS00200	Hypothetical protein
	FGL78_RS00205	Phage tail tape measure protein
	FGL78_RS00215	Hypothetical protein
	FGL78_RS00220	Phage tail protein
	FGL78_RS00225	Hypothetical protein
	FGL78_RS00230	HK97 gp10 family phage protein
	FGL78_RS00235	Hypothetical protein
	FGL78_RS00240	Phage gp6-like head-tail connector protein
	FGL78_RS00245	HeH/LEM domain-containing protein
	FGL78_RS00250	Phage major capsid protein
	FGL78_RS00255	HK97 family phage prohead protease
	FGL78_RS00260	Phage portal protein
	FGL78_RS00270	Phage terminase small subunit P27 family
	FGL78_RS00280	Hypothetical protein
	FGL78_RS00285	Hypothetical protein
	FGL78_RS00290	Hypothetical protein
	FGL78_RS00300	Hypothetical protein
	FGL78_RS00315	Hypothetical protein
	FGL78_RS00320	Hypothetical protein
	FGL78_RS00340	Putative HNHc nuclease
	FGL78_RS00345	Single-stranded DNA-binding protein
	FGL78_RS00350	ERF family protein
	FGL78_RS00355	Hypothetical protein
	FGL78_RS00360	Hypothetical protein
	FGL78_RS00365	Helix-turn-helix domain-containing protein
	FGL78_RS00375	YjzC family protein
	FGL78_RS00380	Hypothetical protein

FGL78_RS00390	Hypothetical protein
FGL78_RS00410	DUF4429 domain-containing protein
FGL78_RS00415	Site-specific integrase
FGL78_RS00420	DUF4065 domain-containing protein
FGL78_RS00430	DUF308 domain-containing protein
FGL78_RS00455	RusA family crossover junction endodeoxyribonuclease
FGL78_RS00460	DUF5906 domain-containing protein
FGL78_RS00465	Hypothetical protein
FGL78_RS00470	DUF669 domain-containing protein
FGL78_RS00475	AAA family ATPase
FGL78_RS00480	Hypothetical protein
FGL78_RS00485	Hypothetical protein
FGL78_RS00490	Hypothetical protein
FGL78_RS00500	Hypothetical protein
FGL78_RS00505	Hypothetical protein
FGL78_RS00610	Hypothetical protein
FGL78_RS00615	Helix-turn-helix transcriptional regulator
FGL78_RS00620	Hypothetical protein
FGL78_RS00625	Hypothetical protein
FGL78_RS00630	Hypothetical protein
FGL78_RS00635	Abi family protein
FGL78_RS00640	Site-specific integrase
FGL78_RS00650	Type II toxin-antitoxin system MqsR family toxin
FGL78_RS00655	DUF4065 domain-containing protein
FGL78_RS01145	Hypothetical protein
FGL78_RS01225	Acyltransferase family protein
FGL78_RS01230	Oligosaccharide flippase family protein
FGL78_RS01235	Polysaccharide pyruvyl transferase family protein
FGL78_RS01240	Glycosyltransferase
FGL78_RS01250	Hypothetical protein
FGL78_RS01725	Hypothetical protein
FGL78_RS01795	Hypothetical protein
FGL78_RS02110	Hypothetical protein
FGL78_RS02215	DNA replication initiation control protein YabA
FGL78_RS02220	IS3 family transposase
FGL78_RS02230	FAD-dependent oxidoreductase
FGL78_RS02235	FAD:protein FMN transferase
FGL78_RS02240	FMN-binding protein
FGL78_RS02865	Hypothetical protein
FGL78_RS02870	Helicase-related protein
FGL78_RS02875	DEAD/DEAH box helicase
FGL78_RS02880	DEAD/DEAH box helicase
FGL78_RS02890	Hypothetical protein
FGL78_RS02900	Hypothetical protein
FGL78_RS02905	AAA family ATPase
FGL78_RS03465	IS3 family transposase
FGL78_RS03920	Hypothetical protein
FGL78_RS04110	Hypothetical protein
FGL78_RS04115	Hypothetical protein
FGL78_RS04120	Protein-export chaperone SecB
FGL78_RS04125	Hypothetical protein
FGL78_RS04130	Helix-turn-helix transcriptional regulator
FGL78_RS04135	Helix-turn-helix transcriptional regulator
FGL78_RS04140	Virulence-associated E family protein
FGL78_RS04145	Hypothetical protein
FGL78_RS04150	Hypothetical protein
FGL78_RS04155	DUF722 domain-containing protein

FGL78_RS04160	HNH endonuclease signature motif containing protein
FGL78_RS04170	Terminase large subunit
FGL78_RS04180	HK97 family phage prohead protease
FGL78_RS04185	Phage major capsid protein
FGL78_RS04190	Head-tail connector protein
FGL78_RS05290	GNAT family protein
FGL78_RS05330	DNA replication initiation control protein YabA
FGL78_RS05335	IS3 family transposase
FGL78_RS07165	Hypothetical protein
FGL78_RS07245	RusA family crossover junction endodeoxyribonuclease
FGL78_RS07250	Helix-turn-helix transcriptional regulator
FGL78_RS07260	Hypothetical protein
FGL78_RS07265	Hypothetical protein
FGL78_RS07270	DUF722 domain-containing protein
FGL78_RS07280	DUF4747 family protein
FGL78_RS07285	Hypothetical protein
FGL78_RS07295	Hypothetical protein
FGL78_RS07385	Hypothetical protein
FGL78_RS07390	Hypothetical protein
FGL78_RS07395	Hypothetical protein
FGL78_RS07400	Hypothetical protein
FGL78_RS07405	Lytic exoenzyme target recognition domain-containing protein
FGL78_RS07410	CsbD family protein
FGL78_RS07415	Y-family DNA polymerase
FGL78_RS07420	Hypothetical protein
FGL78_RS07425	Hypothetical protein
FGL78_RS07665	IS30 family transposase
FGL78_RS08105	MetQ/NlpA family ABC transporter substrate-binding protein
FGL78_RS08125	GNAT family N-acetyltransferase
FGL78_RS08140	IS30 family transposase
FGL78_RS08305	Hypothetical protein
FGL78_RS08335	Recombinase family protein
FGL78_RS08420	IS6 family transposase
FGL78_RS08435	Recombinase family protein
FGL78_RS08440	Helix-turn-helix transcriptional regulator
FGL78_RS08445	NAD(P)H-binding protein
FGL78_RS08465	O-acetylhomoserine aminocarboxypropyltransferase/cysteine synthase
FGL78_RS08515	Hypothetical protein
FGL78_RS08530	Helix-turn-helix transcriptional regulator
FGL78_RS08610	IS30 family transposase
FGL78_RS08625	Hypothetical protein
FGL78_RS08645	IS30 family transposase
FGL78_RS08655	Hypothetical protein
FGL78_RS08680	YhgE/Pip domain-containing protein
FGL78_RS08690	Replication initiation factor domain-containing protein
FGL78_RS08695	Hypothetical protein
FGL78_RS08700	IS30 family transposase
FGL78_RS08950	Hypothetical protein
FGL78_RS09015	DNA replication initiation control protein YabA
FGL78_RS09020	IS3 family transposase
FGL78_RS09235	Glucose-6-phosphate isomerase
FGL78_RS09240	Hypothetical protein
FGL78_RS09245	Asp23/Gls24 family envelope stress response protein
FGL78_RS09250	DUF2273 domain-containing protein
FGL78_RS09255	Alkaline shock response membrane anchor protein AmaP
FGL78_RS09260	GlsB/YeaQ/YmgE family stress response membrane protein

	FGL78_RS09275	IS3 family transposase
	FGL78_RS09280	Hypothetical protein
	FGL78_RS09290	Hypothetical protein
	FGL78_RS09300	IS30 family transposase
	FGL78_RS09305	MFS transporter
	FGL78_RS09310	Pyridoxamine 5-phosphate oxidase family protein
	FGL78_RS09315	Hypothetical protein
	FGL78_RS09345	PII-type proteinase
	FGL78_RS09350	Peptidylprolyl isomerase
	FGL78_RS09360	Hypothetical protein
	FGL78_RS09365	Polysaccharide deacetylase family protein
	FGL78_RS09375	DDE-type integrase/transposase/recombinase
	FGL78_RS09380	Integrase core domain-containing protein
	FGL78_RS09390	Hypothetical protein
	FGL78_RS09470	DDE-type integrase/transposase/recombinase
	FGL78_RS09475	Integrase core domain-containing protein
	FGL78_RS09490	Integrase core domain-containing protein
	FGL78_RS09495	ABC transporter permease
	FGL78_RS09500	ATP-binding cassette domain-containing protein
	FGL78_RS09505	NEAT domain-containing protein
	FGL78_RS09510	NEAT domain-containing protein
	FGL78_RS09540	Hypothetical protein
	FGL78_RS09550	Hypothetical protein
	FGL78_RS09560	Hypothetical protein
	FGL78_RS09570	Hypothetical protein
	FGL78_RS09600	Hypothetical protein
	FGL78_RS09615	Biotin carboxylase N-terminal domain-containing protein
	FGL78_RS09630	IS3 family transposase
	FGL78_RS09645	Hypothetical protein
	FGL78_RS09655	Helix-turn-helix domain-containing protein
	FGL78_RS09665	Hypothetical protein
	FGL78_RS09670	Hypothetical protein
	FGL78_RS09675	Hypothetical protein
	FGL78_RS09680	Hypothetical protein
	FGL78_RS09685	Helix-turn-helix transcriptional regulator
	FGL78_RS09690	Hypothetical protein
	FGL78_RS09695	Hypothetical protein
	FGL78_RS09705	Hypothetical protein
	FGL78_RS09715	FAD-dependent oxidoreductase
	FGL78_RS09735	Hypothetical protein
	FGL78_RS09745	Hypothetical protein
	FGL78_RS09755	Hypothetical protein
	FGL78_RS09760	Transposase
	FGL78_RS09765	Transposase family protein
WiKim40	BCR17_RS00095	Helix-turn-helix domain-containing protein
	BCR17_RS00315	IS30 family transposase
	BCR17_RS00320	Helix-turn-helix domain-containing protein
	BCR17_RS00325	Hypothetical protein
	BCR17_RS00330	Ion transporter
	BCR17_RS00350	Hypothetical protein
	BCR17_RS00375	Hypothetical protein
	BCR17_RS00390	Helix-turn-helix transcriptional regulator
	BCR17_RS00495	IS3 family transposase
	BCR17_RS00500	Transposase
	BCR17_RS00660	IS3 family transposase
	BCR17_RS00665	Helix-turn-helix domain-containing protein
	BCR17_RS01250	IS3 family transposase

BCR17_RS01360	Hypothetical protein
BCR17_RS01710	Transposase
BCR17_RS02435	Helix-turn-helix domain-containing protein
BCR17_RS02440	IS3 family transposase
BCR17_RS03175	Transposase
BCR17_RS03380	IS3 family transposase
BCR17_RS03385	Transposase
BCR17_RS03630	Iron ABC transporter permease
BCR17_RS03635	ABC transporter ATP-binding protein
BCR17_RS03640	Extracellular solute-binding protein
BCR17_RS03810	Aldo/keto reductase
BCR17_RS03815	VOC family protein
BCR17_RS04230	DUF1819 family protein
BCR17_RS04235	DUF1788 domain-containing protein
BCR17_RS04240	BREX system P-loop protein BrxC
BCR17_RS04245	BREX-1 system adenine-specific DNA-methyltransferase PglX
BCR17_RS04255	BREX-1 system adenine-specific DNA-methyltransferase PglX
BCR17_RS04260	BREX-1 system phosphatase PglZ type A
BCR17_RS04265	Protease Lon-related BREX system protein BrxL
BCR17_RS04915	AAA family ATPase
BCR17_RS04940	Hypothetical protein
BCR17_RS05515	Restriction endonuclease subunit S
BCR17_RS05520	HsdR family type I site-specific deoxyribonuclease
BCR17_RS05525	Restriction endonuclease subunit S
BCR17_RS05530	Type I restriction-modification system subunit M
BCR17_RS05545	Site-specific integrase
BCR17_RS06210	Hypothetical protein
BCR17_RS06215	Exosortase family protein XrtG
BCR17_RS06220	Putative glycosyltransferase, exosortase G system-associated
BCR17_RS06225	6-carboxytetrahydropterin synthase
BCR17_RS06555	NAD(P)-dependent oxidoreductase
BCR17_RS06565	NDP-hexose 2,3-dehydratase family protein
BCR17_RS06605	Hypothetical protein
BCR17_RS06745	IS3 family transposase
BCR17_RS06750	Transposase
BCR17_RS07170	ParA family protein
BCR17_RS07175	Hypothetical protein
BCR17_RS07180	Hypothetical protein
BCR17_RS07260	Hypothetical protein
BCR17_RS07600	Tryptophan synthase subunit beta
BCR17_RS07605	Anthranilate phosphoribosyltransferase
BCR17_RS07610	Indole-3-glycerol phosphate synthase TrpC
BCR17_RS07620	Tryptophan synthase subunit alpha
BCR17_RS07625	Anthranilate synthase component I
BCR17_RS07630	Aminodeoxychorismate/anthranilate synthase component II
BCR17_RS07690	Acyltransferase
BCR17_RS07825	Potassium-transporting ATPase subunit KdpB
BCR17_RS07915	Hypothetical protein
BCR17_RS07920	Hypothetical protein
BCR17_RS07995	Phage tail tip lysozyme
BCR17_RS08115	DUF5067 domain-containing protein
BCR17_RS08120	DUF3862 domain-containing protein
BCR17_RS08125	Cold-shock protein
BCR17_RS08190	Ribonuclease H
BCR17_RS08195	IS3 family transposase
BCR17_RS08250	Hypothetical protein
BCR17_RS08345	Beta-galactosidase

BCR17_RS08350	LacI family DNA-binding transcriptional regulator
BCR17_RS08370	Hypothetical protein
BCR17_RS08375	DUF916 domain-containing protein
BCR17_RS08385	WxL domain-containing protein
BCR17_RS08390	Class A sortase
BCR17_RS08395	DUF916 and DUF3324 domain-containing protein
BCR17_RS08400	WxL domain-containing protein
BCR17_RS08405	WxL domain-containing protein
BCR17_RS08415	Helix-turn-helix domain-containing protein
BCR17_RS08420	Hypothetical protein
BCR17_RS08445	Alpha/beta hydrolase
BCR17_RS08450	NADP-dependent oxidoreductase
BCR17_RS08455	TetR/AcrR family transcriptional regulator
BCR17_RS08535	MobV family relaxase
BCR17_RS08540	GNAT family N-acetyltransferase
BCR17_RS08545	Glycoside hydrolase family 32 protein
BCR17_RS08550	MFS transporter
BCR17_RS08555	LacI family DNA-binding transcriptional regulator
BCR17_RS08565	Metalloregulator ArsR/SmtB family transcription factor
BCR17_RS08570	NAD(P)/FAD-dependent oxidoreductase
BCR17_RS08575	DsbA family oxidoreductase
BCR17_RS08580	Thioredoxin-disulfide reductase
BCR17_RS08585	Thioredoxin family protein
BCR17_RS08590	Thioredoxin
BCR17_RS08655	MobV family relaxase
BCR17_RS08660	Hypothetical protein
BCR17_RS08670	IS3 family transposase
BCR17_RS08680	Hypothetical protein
BCR17_RS08690	MucBP domain-containing protein
BCR17_RS08695	IS30 family transposase
BCR17_RS08715	EcsC family protein
BCR17_RS08725	Hypothetical protein
BCR17_RS08740	HTH domain-containing protein
BCR17_RS08750	S41 family peptidase
BCR17_RS08790	Serine acetyltransferase
BCR17_RS08835	IS5 family transposase
BCR17_RS08840	Hypothetical protein
BCR17_RS08850	Hypothetical protein
BCR17_RS08865	Hypothetical protein
BCR17_RS08900	Hypothetical protein
BCR17_RS08905	Hypothetical protein
BCR17_RS08960	RTX toxin
BCR17_RS08970	LacI family DNA-binding transcriptional regulator
BCR17_RS08975	Hypothetical protein
BCR17_RS08980	Hypothetical protein
BCR17_RS08990	LPXTG cell wall anchor domain-containing protein
BCR17_RS09000	AAA family ATPase
BCR17_RS09045	Hypothetical protein

Table S2. Qualitative enzyme activity measure in clear zone around filter paper disc containing DMLL10.

Strain	Protease		Lipase	Acid
	0.5%	3%	0.5%	0.5%
DMLL10	2.45±0.07[b]	2.00±0.00[a]	0.00±0.00[a]	1.95±0.07[b]
KCTC 3528[T]	2.05±0.07[b]	0.00±0.00[a]	0.00±0.00[a]	1.80±0.10[c]

Different letter indicates significant difference at $p < 0.05$ using Duncan's multiple range test.

NaCl concentration in TSA is 0.5% (w/v), and the mean values of replicates are presented.

Table S3. Qualitative antibacterial activity measure in clear zone around cork-hole containing DMLL10.

	DMLL10	KCTC 3528^T
Gram-positive		
Bacillus cereus	1.90±0.14^b	1.00±0.00^a
Enterococcus faecalis	1.55±0.07^a	0.15±0.07^a
Listeria monocytogenes	1.10±1.14^c	0.10±0.00^a
Staphylococcus aureus	2.50±0.00^a	1.10±0.14^b
Gram-negative		
Alclaigenes xylosoxidans	0.15±0.07^a	0.00±0.00^a
Escherichia coli O157:H7	0.00±0.00^a	0.00±0.00^a
Flavobacterium sp.	3.00±0.00^a	0.10±0.00^a
Salmonella enterica	3.00±0.00^a	0.15±0.07^a
Vibrio parahaemolyticus	2.90±0.14^b	0.80±0.14^b

Different letter indicates significant difference at $p < 0.05$ using Duncan’s multiple range test.
